# Effect of saline water on seed germination and early seedling growth of the halophyte quinoa

**DOI:** 10.1093/aobpla/plu047

**Published:** 2014-08-19

**Authors:** M. R. Panuccio, S. E. Jacobsen, S. S. Akhtar, A. Muscolo

**Affiliations:** 1 Department of Agriculture, Mediterranea University, località Feo di Vito, 89126 Reggio Calabria, Italy; 2 Department of Plant and Environmental Sciences, Faculty of Science, University of Copenhagen, Højbakkegård Allé 13, DK-2630 Tåstrup, Denmark; 3 Sino-Danish Center for Education and Research (SDC), Beijing, China

**Keywords:** Abiotic stress, antioxidant enzymes, *Chenopodium quinoa*, germination, root morphology, salinity stress, seedling growth

## Abstract

Salinization is increasing on a global scale, decreasing average yields for most major crop plants. Investigations into salt resistance have, unfortunately, mainly been focused on conventional crops, with few studies screening the potential of available halophytes as new crops. This study has been carried out to investigate the mechanisms used by quinoa, a facultative halophytic species, in order to cope with high salt levels at various stages of its development. Quinoa is regarded as one of the crops that might sustain food security in this century, grown primarily for its edible seeds with their high protein content and unique amino acid composition. Although the species has been described as a facultative halophyte, and its tolerance to salt stress has been investigated, its physiological and molecular responses to seawater (SW) and other salts have not been studied. We evaluated the effects of SW and different salts on seed germination, seedling emergence and the antioxidative pathway of quinoa. Seeds were germinated in Petri dishes and seedlings grown in pots with SW solutions (25, 50, 75 and 100 %) and NaCl, CaCl_2_, KCl and MgCl_2_ individually, at the concentrations in which they are present in SW. Our results demonstrated that all salts, at lower concentrations, increased the germination rate but not the germination percentages, compared with control (pure water). Conversely, seedlings were differently affected by treatments in respect to salt type and concentration. Growth parameters affected were root and shoot length, root morphology, fresh and dry weight, and water content. An efficient antioxidant mechanism was present in quinoa, activated by salts during germination and early seedling growth, as shown by the activities of antioxidant enzymes. Total antioxidant capacity was always higher under salt stress than in water. Moreover, osmotic and ionic stress factors had different degrees of influence on germination and development.

## Abstract (precis)

The introduction of new crops with improved salinity stress tolerance could preserve water quality and protect soil resources from further degradation, providing extra sources of food for salinized areas. In this context, we tested the salinity tolerance of a variety of quinoa. Quinoa, a rich source of minerals, proteins and antioxidants, is considered a major alternative crop to meet food shortages in this century. Our study indicated that salinity tolerance of quinoa is largely conferred by a delicate balance between osmotic adjustment and ion accumulation. Salinity reduced productivity in terms of biomass, but increased the levels of antioxidant compounds, which are important health-protecting factors in food, thus providing economic benefit.

## Introduction

Soil salinity and sodicity cause severe problems in agriculture worldwide, and salt tolerance in crops is an extremely important trait and a major focus of research. Detrimental effects of high salinity on crops are multifaceted and affect plants in several ways: drought stress, ion toxicity, nutritional disorders, oxidative stress, alteration of metabolic processes, membrane disorganization and reduction of cell division and expansion (Hasegawa *et al.* 2000; Munns 2002; Muscolo *et al.* 2007, 2013; Zhu 2007; Sidari *et al.* 2008). As a result, plant growth, development and survival are reduced (Muscolo *et al.* 2011; Schleiff and Muscolo 2011). Two major stresses affecting plants under salinity are osmotic and ionic stresses. Osmotic stress, occurring immediately in the root medium on exposure to salts, can result in inhibition of water uptake, cell expansion and lateral bud development (Munns and Tester 2008). Ionic stress develops when toxic ions (e.g. Na^+^) accumulate in cells causing increase in leaf mortality, chlorosis, necrosis and decrease in the activity of cellular metabolism including photosynthesis (Yeo and Flowers 1986; Glenn and Brown 1999). In fact, excess Na^+^ and Cl^−^ have the potential to affect plant enzymes, resulting in reduced energy production and other physiological processes (Larcher 1980; Morais *et al.* 2012*a**,**b*). Ionic stress results in premature senescence of older leaves and in toxicity symptoms (chlorosis, necrosis) in mature leaves due to high Na^+^ and Cl^−^ which affect plants by disrupting protein synthesis and by interfering with enzyme activity (Munns and Termaat 1986; Hasegawa *et al.* 2000; Munns 2002).

In order to counteract the detrimental effects of salinity on agricultural production, extensive research on plant screening for salt tolerance has been conducted, with the aim of providing more tolerant cultivars. However, these studies have mainly focused on conventional crops, screening criteria and investigating how plants tolerate salts (Shannon and Noble 1990; Chen *et al.* 2005; Sevengor *et al.* 2011). Unfortunately, there are few investigations on screening of available halophytes and their responses to saline conditions (Flowers *et al.* 2010). The seed crop quinoa is a facultative halophyte native to the Andean region of Bolivia and Peru, and a member of the Amaranthaceae: quinoa is traditionally cultivated across a range of extreme environments. Due to its huge genetic variability, the species can be grown under unfavourable soil and climatic conditions (Ruiz-Carrasco *et al.* 2011), showing a diverse tolerance to a wide range of abiotic stresses such as frost, salinity and drought, as well as an ability to grow on marginal soils (Jacobsen *et al.* 2005, 2007, 2009; Maughan *et al.* 2009; Sun *et al.* 2014). Some varieties can grow in salt concentrations similar to those found in seawater (SW, 40 dS m^−1^) and even higher (Jacobsen *et al.* 2001; Adolf *et al.* 2012, 2013; Shabala *et al.* 2012, 2013), well above the threshold for any known crop species.

Quinoa is considered a major alternative crop to meet food shortages in this century (Jensen *et al.* 2000; Jacobsen *et al.* 2003; Sanchez *et al.* 2003; Trognitz 2003; Ruiz *et al.* 2014), for its gluten-free seeds and also as its grains provide a rich source of a wide range of minerals (Ca, P, Mg, Fe and Zn), vitamins (B_1_, B_9_, C and E), linolenate, natural antioxidants and high-quality protein, containing ample amounts of essential amino acids such as lysine and methionine (Abugoch *et al.* 2008; Koyro and Eisa 2008). Quinoa's tolerance to high salinity at the primary stages of seed germination is based upon alterations in the levels of primary metabolites and enzyme activity (González and Prado 1992; Adolf *et al.* 2013). Most of the studies on the effect of salinity on seed germination of halophytes have, however, been conducted using NaCl solutions. Such investigations may not provide information on germination under field conditions, because soils contain different salts, which may collectively influence germination in different ways from their individual effects (Ungar 1996). Sea salt mimics the composition of saline soil solutions and can be used to study the synergistic effect of different salts on seed germination (Liu *et al.* 2006). Therefore, the work presented here was carried out to examine the effects of SW and its component salts on seed germination, seedling emergence and the antioxidative pathway of quinoa cv. Titicaca, as well as the relative importance of two components (ionic and osmotic) of salinity stress.

Quinoa cultivars have been shown to differ in salt tolerance (Bonales-Alatorre *et al.* 2013). In general, varieties originating from salt-affected areas are adapted to saline conditions and hence are less affected by salinity (Adolf *et al.* 2012; Shabala *et al.* 2013) than those from non-saline areas. In this study, we used the Danish-bred quinoa cv. Titicaca (Jacobsen *et al.* 2010; Adolf *et al.* 2012) to verify the salinity tolerance of a variety well adapted to European climatic conditions. Quinoa production may be a viable option for farmers interested in a high-value crop with regional production and local markets in Mediterranean countries where saline water and soil salinity are major risks for the future of agricultural development. Here fresh water resources are limited, while food requirements and pressure from climate change are still growing. The use of saline water resources may constitute a remedy for the current water scarcity. For these reasons, quinoa offers the possibility of an alternative, promising, cash crop to be cultivated in arid and semiarid environments that are prohibitive for other species and so may be able to utilize saline soils in a sustainable and productive way.

## Methods

### Plant material

Mature seeds of the Danish-bred quinoa (*Chenopodium quinoa* cv. Titicaca) (provided by Department of Plant Environmental Science, University of Copenhagen) were stored at 5 °C until the start of experiments. Two different experiments were carried out in a growth chamber (Green line WRS 96-85, KW, Scientific Equipment, Italy) (temperature of 25 ± 1 °C in the dark with a relative humidity of 70 %) to characterize the responses of quinoa to salt stress. Seed germination and biochemical responses were studied in the first experiment, while morphological, physiological and biochemical responses of seedlings were studied in the second experiment.

### Experiment 1: seed germination

#### Germination conditions and experimental design

Seeds were surface-sterilized for 20 min in 20 % (v/v) sodium hypochlorite, rinsed and soaked for 1 h in distilled water. The sterilization procedure is required to eliminate saponine from seeds and to avoid contamination by microorganisms during the germination process. The entire sterilization procedure, including soaking, took 1 h and did not affect the germination process (Ruiz-Carrasco *et al.* 2011; Burrieza *et al.* 2012). For the germination tests, five 50-seed replicates were used with either Mediterranean SW collected from the Tirreno sea (Calabria Southern Italy) with a salinity of 38 % (Cotruvo 2005) or solutions of NaCl, CaCl_2_, KCl or MgCl_2_ at the concentration in which they were in the SW and at various dilutions. In the experiment, five different concentrations of NaCl (0, 100, 200, 300 and 400 mM); KCl (0, 2.54, 5.08, 7.62 and 10.2 mM); CaCl_2_ (0, 2.54, 5.08, 7.62 and 10.2 mM) and MgCl_2_ (0, 13.4, 26.7, 40.1 and 53.5 mM) were used to test whether the various ions differently affected germination indexes and to verify possible antagonistic or synergic ion effects on seed germination. Seeds were placed on filter paper in 9 cm diameter Petri dishes containing 3 mL of each solution. The Petri dishes were hermetically sealed with Parafilm to prevent evaporation and kept in the growth chamber at a temperature of 25 ± 1 °C in the dark with a relative humidity of 70 %. Seeds were considered germinated when the radicle had extended at least 2 mm.

#### Germination indexes

The number of seeds germinated was recorded daily for up to 7 days. From these germination counts, several germination attributes were calculated to characterize the salt tolerance, including germination percentage (%) at 1 and 7 days, coefficient of velocity of germination (CVG) (Kader and Jutzi 2004), germination rate index (GRI) (Kader 2005) and mean germination time (MGT) (Kader 2005), as follows:

$$\begin{array}{rr} & \text{CVG}\;(\%\;{\text{day}}^{-1})=\sum N_{i}/\sum (N_{i}T_{i})\times 100,\\ & \text{GRI}\;(\%\;{\text{day}}^{-1})=\sum N_{i}{/}_{I},\\ & \text{MGT}(\text{days})=\sum (N_{i}T_{i})/\sum N_{i} \end{array}$$

where *N* is the number of seeds germinated on day *i*, and *T_i_* is the number of days from sowing. The CVG gives an indication of the rapidity of germination: it increases when the number of germinated seeds increases and the time required for germination decreases. The GRI reflects the percentage of germination on each day of the germination period. Higher GRI values indicate higher and faster germination. The lower the MGT, the faster a population of seeds has germinated.

#### Determination of ionic and osmotic effect

According to Munns *et al.* (1995), the decrease in germination under saline conditions is the consequence of the combined effect of osmotic (OE) and ionic (IE) factors; consequently, the total effect (TE) of salinity on germination can be defined as

TE = OE + IE

To resolve this equation, the osmotic components (OE) were determined by germinating seeds in distilled water (zero osmolality) and in solutions of polyethylene glycol (PEG 8000) with an osmolality equivalent to the concentrations of the various salts that reduced germination by 50 % (LD_50max_). Consequently, OE corresponds to the difference between the germination values obtained in pure water (GH_2_O) and those obtained in the isotonic solutions (GOs).

$${\text{OE = GH}}_{2}\text{O}-\text{GOs}$$

A cryoscopic osmometer (OSMOMAT 030 GONOTEC) was used in order to determine the osmolality of each PEG and saline solution. The TE of salinity was obtained by means of the difference between the germination values under non-saline conditions with water (GH_2_O) and germination obtained with the LD_50max_ saline concentrations. This germination is termed GLD_50max_, thus TE was determined through:

$${\text{TE = GH}}_{2}\text{O}-{\text{GLD}}_{50\max}$$

Based on the values of TE and OE, the ionic effect (IE) was calculated as

IE = TE−OE

#### Determination of enzyme activities

Seeds (0.5 g) that had been soaked for 3 days in the test solutions were ground using a chilled mortar and pestle and homogenized in 0.1 M phosphate buffer solution (pH 7.0) containing 100 mg soluble polyvinylpolypyrrolidone and 0.1 mM ethylenediamine tetra acetic acid (EDTA). The homogenate was filtered through two layers of muslin cloth and centrifuged at 15 000 *g* for 15 min at 4 °C. The resulting supernatant was used to evaluate the activity of catalase (CAT, EC 1.11.1.6), peroxidase (POX, EC 1.11.1.7), ascorbate peroxidase (APX, EC 1.11.1.11) and superoxide dismutase (SOD EC 1.15.1.1). All enzyme activities were measured at 25 °C by a UV–visible light spectrophotometer (UV-1800 CE, Shimadzu, Japan).

Catalase activity was determined by monitoring the disappearance of H_2_O_2_ at 240 nm, calculated using its extinction coefficient (*ε*) = 0.036 mM^−1^ cm^−1^. The reaction mixture contained 1 mL of potassium phosphate buffer (50 mM, pH 7.0), 40 μL of enzyme extract and 5 μL of H_2_O_2_ (Beaumont *et al.* 1990).

Ascorbate peroxidase activity was assayed according to Nakano and Asada (1981). The reaction mixture (1.5 mL) contained 50 mM phosphate buffer (pH 6.0), 0.1 μM EDTA, 0.5 mM ascorbate, 1.0 mM H_2_O_2_ and 50 μL enzyme extract. The reaction was started by the addition of H_2_O_2_ and ascorbate oxidation measured at 290 nm for 1 min. Enzyme activity was quantified using the molar extinction coefficient for ascorbate (2.8 mM^−1^ cm^−1^).

Peroxidase activity was measured on the basis of determination of guaiacol oxidation at 436 nm for 90 s (Panda *et al.* 2003). The reaction mixture contained 1 mL of potassium phosphate buffer (0.1 M, pH 7.0), 20 μL of guaiacol, 40 μL of enzyme extract and 15 μL of H_2_O_2_. Peroxidase activity was quantified by the amount of tetraguaiacol formed using its extinction coefficient (*ε*) = 25.5 mM^−1^ cm^−1^.

Superoxide dismutase activity was estimated by recording the decrease in the absorbance of superoxide nitro-blue tetrazolium complex by the enzyme (Gupta *et al.* 1993). The reaction mixture (3 mL) contained 0.1 mL of 200 mM methionine, 01 mL of 2.25 mM nitro-blue tetrazolium, 0.1 mL of 3 mM EDTA, 1.5 mL of 100 mM potassium phosphate buffer, 1 mL of distilled water and 0.05 mL of enzyme extract. The assay was performed in duplicate for each sample. Two tubes without enzyme extract were used as a background control. The reaction was started by adding 0.1 mL of riboflavin (60 μM) and placing the tubes below a light source of two 15 W florescent lamps for 15 min. The reaction was stopped by switching off the light and covering the tubes with black cloth. Tubes without enzyme developed maximum colour. A non-irradiated complete reaction mixture which did not develop colour served as the blank. Absorbance was recorded at 560 nm and one unit of enzyme activity was taken as the quantity of enzyme which reduced the absorbance of samples to 50 % in comparison with tubes lacking enzymes.

For CAT, APX, SOD and POX activities, the results were expressed as enzyme units (U) per mg fresh weight. One unit of enzyme was defined as the amount of enzyme necessary to decompose 1 μmol of substrate per min at 25 °C.

#### Determination of total antioxidant capacity

Seeds (treated with different salt solutions for 3 days) were homogenized in a chilled mortar with distilled water at a ratio of 1 : 4 (seeds/water; w/v) and centrifuged at 14 000 *g* for 30 min. All steps were performed at 4 °C. The supernatants were filtered through two layers of muslin cloth and were used to determine the total antioxidant capacity by the spectrophotometric method of Prieto *et al.* (1999). Aqueous extracts of the seeds were mixed in Eppendorf tubes with 1 mL of reagent solution (0.6 M H_2_SO_4_, 28 mM sodium phosphate, 4 mM ammonium molybdate mixture). The tubes were incubated for 90 min at 95 °C, then cooled to room temperature, and the absorbance read at 695 nm against a blank (mixture without seed extract). The assay was conducted in triplicate and the total antioxidant activity expressed as the absorbance of the sample at 695 nm. The higher the absorbance value, the higher the antioxidant activity (Prasad *et al.* 2009).

#### Determination of total phenolic content

Total phenolic content was determined with the Folin–Ciocalteu reagent according to a modified procedure described by Singleton and Rossi (1965). Briefly, 0.50 mL of the aqueous extract of the seeds was reacted with 2.5 mL of Folin–Ciocalteu reagent (1 : 10 diluted with distilled water) for 4 min, and then 2 mL of saturated sodium carbonate solution (∼75 g/L) was added to the reaction mixture. The absorbance readings were taken at 760 nm after incubation at room temperature for 2 h. Tannic acid was used as a reference standard, and the results were expressed as milligram tannic acid equivalent (mg TAET/g fresh weight).

### Experiment 2: morphological, physiological and biochemical responses of seedlings

#### Plantlet growth in pots

Seeds were germinated in Petri dishes. After 3 days from the beginning of germination, germinated seeds were grown for 21 days in plastic pots (10 cm diameter × 7 cm height), in a growth chamber (Green line WRS 96-85, KW apparecchi scientifici, Italy), under white light (80 W m^−2^, Osram HQI halogen vapor W lamp, PAR 1055 μmol m^−2^ s−1) in a 16/8-h photoperiod, 70 % relative humidity and at 21 °C. All pots were filled with Perlite that had been equilibrated, before transplanting the germinated seeds, with one of the different salts or SW solutions at the desired concentration. All reagents used were of the highest analytical grade and were purchased from Sigma Chemical Co. (St. Louis, MO, USA). All pots were watered with a one-fourth strength Murashige and Skoog medium (MS_/4_) containing macro and micronutrients at pH 5.8: the pots were weighed daily, and watered when their weight decreased by 30 % (corresponding to water that was lost by evapotranspiration). The control pots were watered with MS_/4_ alone. Leaf and root length were evaluated 21 days after the beginning of the stress, using six plants for each treatment.

#### Measurement of enzyme activities

After 21 days in pots under different salinity treatments, plantlet material was ground with a mortar and pestle in 100 mM HEPES–NaOH (pH 7.5), 5 mM MgCl_2_ and 1 mM dithiothreitol . The ratio of plant material to buffer was 1 : 3. The extract was filtered through two layers of muslin and clarified by centrifugation at 15 000 *g* for 15 min. The supernatant was used for CAT, APX, POX, SOD analyses and total antioxidant capacity as described above. All steps were performed at 4 °C.

Cations (Na^+^, K^+^, Ca^2+^ Mg^2+^ and NH_4_^+^) and anions (Cl^−^ and SO_4_^2−^) were determined in the water extracts of treated seedlings by ion chromatography (DIONEX ICS-1100).

#### Measurement of root morphology

Seedlings were harvested and root weight was recorded. Roots were scanned using an Epson Expression/STD 1600 scanner and personal computer with Intel Pentium III/500 CPU, 128 MB RAM, optimized for root analyses by Regent Instrument, Inc., and their length was analysed using the WinRHIZO image analysis system (Regent Instruments, Quebec, Canada). When scanning, each root sample was placed in a rectangular glass dish (300 × 200 mm) with ∼4–5 mm of water to untangle the roots and minimize root overlap. Three replicated roots were analysed for each treatment.

### Statistical analysis

All data were analysed by one-way analysis of variance (ANOVA) with the salt concentration as the grouping factor. Separate ANOVAs were performed for each of four salt types and concentrations: NaCl (0, 100, 200, 300, 400 mM); KCl (0, 2.54, 5.08, 7.62, 10.16 mM); CaCl_2_ (0, 2.54, 5.08, 7.62, 10.16 mM) and MgCl_2_ (0, 13.36, 26.72, 40.09, 53.46 mM). The response variables for these ANOVAs were: seed germination, seedling growth, enzyme activities, ion contents and root morphology. Since salt concentration had five levels, on all significant ANOVAs we performed Tukey's multiple comparison tests to compare all pairs of means. The germination percentage data were previously subjected to arcsine transformation but are reported in tables as untransformed values. All data collected were statistically analysed using SYSTAT 8.0 software (SPSS Inc.).

## Results

### Experiment 1: Germination under saline conditions

In water, all (100 %) seeds germinated (Table 1). At the lower concentrations, individual salts (NaCl, CaCl_2_, KCl and MgCl_2_) did not have any significant effects on the germination percentage of quinoa seeds. Conversely, dilute SW significantly lowered germination (Table 1). With increasing salt concentration, the germination percentage decreased, irrespective of the treatment, except for MgCl_2_. The strongest reduction of germination was observed in the presence of 75 and 100 % SW in comparison to the other salts. The inhibition of different salt solutions on seed germination was in the order of SW > NaCl > KCl > CaCl_2_ > MgCl_2_ (Table 1). There were no significant differences among the treatments in germination rapidity (CVG), except in the SW (Table 1: with increasing SW concentration, the CVG decreased, with a reduction of 53 % at 75 % SW). The GRI, reflecting the percentage of germination on each day of the germination period, decreased under NaCl and SW. The strongest decrease was observed in SW. No significant differences were observed among NaCl, CaCl_2_, KCl and MgCl_2_ and the control, in terms of MGT (MGT, Table 1). Conversely, with increasing SW percentage, the MGT increased, reaching values 10 times greater than the control and of the other treatments. The strong significant inverse relationship between SW concentrations and germination indexes confirmed the detrimental effects of the SW on seed germination (Table 1).

**Table 1.** Germination indices: total germination; CVG, GRI and MGT determined for quinoa seeds after 7 days of germination in the presence of NaCl, CaCl_2_, KCl, MgCl_2_ and SW at different concentrations. Data are expressed as percentage in respect to control. Data are the means of five replicates. ***P < 0.001; **P < 0.01: *P < 0.05.

		Total germination (%)	CVG (%)	GRI (%)	MGT (days)
Control		100	26.8	26.7	3.7
NaCl	100 mM	100	26.2	26.4	3.8
NaCl	200 mM	100	27.0	28.4	3.7
NaCl	300 mM	95	26.0	24.7*	3.8
NaCl	400 mM	80**	26.7	22.2*	3.7
KCl	2.54 mM	96	26.8	26.7	3.8
KCl	5.08 mM	95	26.8	26.7	3.8
KCl	7.62 mM	93*	26.5	25.3	3.8
KCl	10.16 mM	86*	26.9	23.9*	3.7
MgCl_2_	13.36 mM	100	26.1	26.1	3.7
MgCl_2_	26.73 mM	100	26.9	28.3	3.7
MgCl_2_	40.00 mM	100	26.3	26.6	3.7
MgCl_2_	53.46 mM	100	27.0	29.1*	3.6
CaCl_2_	2.54 mM	98	26.6	26.8	3.8
CaCl_2_	5.08 mM	95	26.4	25.4	3.8
CaCl_2_	7.62 mM	93*	26.4	24.9	3.8
CaCl_2_	10.16 mM	93*	27.0	26.8	3.7
SW	25 %	85*	25.8*	21.4**	3.9
SW	50 %	65***	19.6**	6.6***	5.1*
SW	75 %	10***	14.3**	0.28***	35***
SW	100 %	0	nd	nd	nd

#### Separation of ionic and osmotic components

Calculating the relative importance of the osmotic and ionic component stresses showed that the two stressful factors made a different contribution to the deterioration of germination depending on the salts used. In the presence of MgCl_2_, the two stressful factors (ionic and osmotic) had a proportional effect on the reduction of seed germination as shown by the value of the IE/OE ratio (1.0, Table 2). Regarding NaCl, the osmotic effect prevailed (IE/OE ratio = 0.53). In CaCl_2_ and KCl, at LD_50_ concentrations, seed germination decreased, mainly due to osmotic factors, as suggested by the IE/OE ratios that were always <1.0 and by IE values that were under 50 (Table 2). Seawater (the most toxic) affected seed germination mainly through its IE as evidenced by the IE/OE ratio >1.0 (Table 2).

**Table 2.** Influence of osmotic and ionic factors on seed germination of Titicaca quinoa seeds in the presence of NaCl, KCl, MgCl_2_, CaCl_2_ and SW at LD_50max_ concentration. *Different letters in the same column denote significant differences among treatments (*P* ≤ 0.05). The values correspond to the average of five replicates.

Treatments	OE	TE (OE + IE) (%)	IE	IE/OE	IE/TE
NaCl	36^a^	55	19^c^	0.53^d^	35^d^
KCl	29^b^	52	23^b^	0.79^c^	44^c^
MgCl_2_	27^b^	54	27^b^	1.0^b^	50^b^
CaCl_2_	33^a^	58	25^b^	0.76^c^	43^c^
SW	20^c^	60	40^a^	2.0^a^	67^a^

#### Enzyme activities, phenols and antioxidants

**Figure 1.**
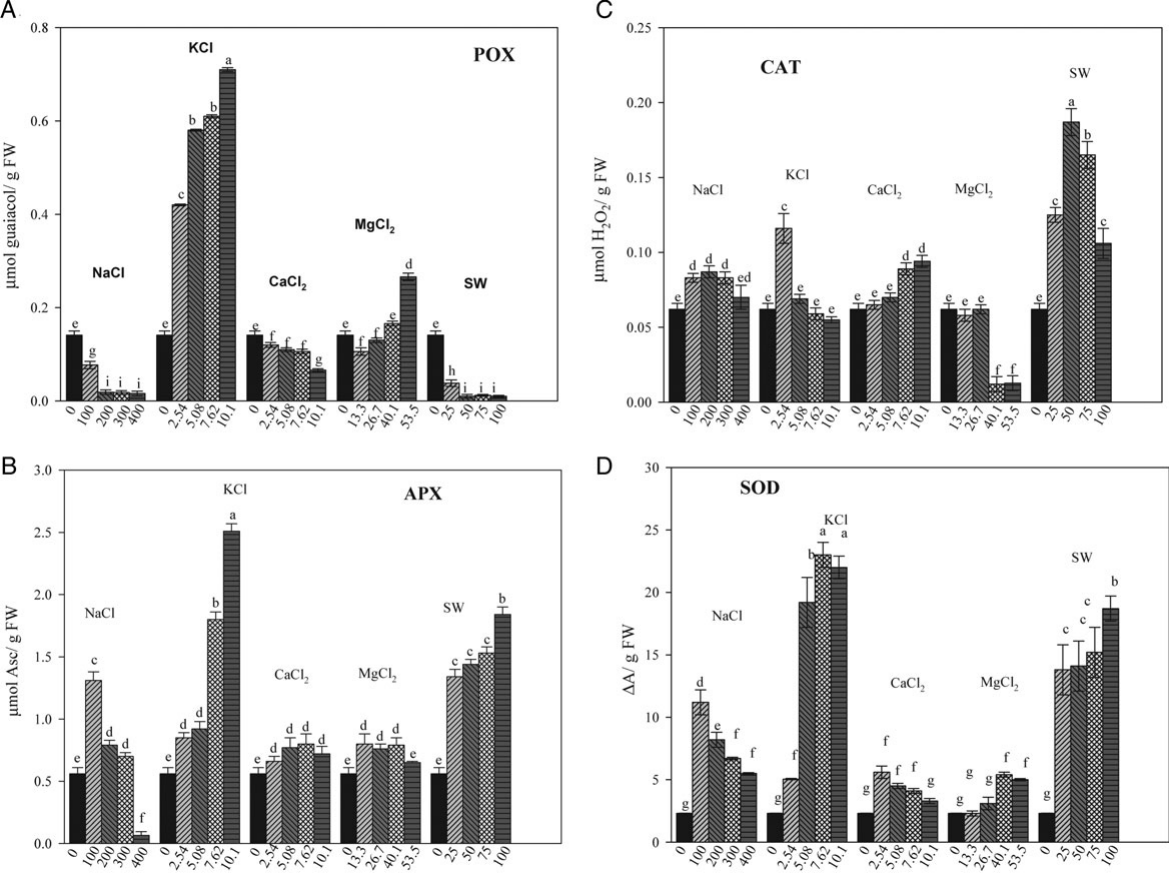
Effect of different salts on POX, APX, CAT, SOD of quinoa seeds 3 days after sowing. Mean ± SE (*n* = 4–5). Different letters denote significant differences among the treatments (*P* ≤ 0.05).

With increasing salt concentrations, POX activity decreased, with respect to the control in the presence of NaCl, CaCl_2_ and SW. Conversely, an increase in POX activity was observed with MgCl_2_ and particularly KCl (Fig. 1A). Ascorbate peroxidase, CAT and SOD activities were always lower in control seeds compared with treated seeds; the highest concentrations of KCl and SW increased APX activity five and four times, respectively, compared with control. In NaCl and MgCl_2_, APX activity was higher at the lower, than at the higher, concentrations, and it was unaffected by CaCl_2_ treatment (Fig. 1B). Catalase activity increased with increasing concentration of CaCl_2_ and SW. In contrast, in the presence of KCl and MgCl_2_, CAT activity decreased when the concentration increased (Fig. 1C). Superoxide dismutase activity decreased as the concentrations of NaCl and CaCl_2_ increased. Conversely, in the presence of increasing concentrations of KCl, MgCl_2_ and SW, SOD activity increased, but to different extents. The highest values of SOD were observed in the presence of SW and KCl (Fig. 1D).

The amount of total phenols and the total antioxidant capacity of seeds varied with the salt used. Total phenols increased in seeds treated with NaCl and SW, but the greatest increase was observed in the presence of SW (Table 3). Increasing the concentrations of KCl and MgCl_2_ decreased total phenols; no significant differences were instead observed with increasing  the concentration of CaCl_2_ with respect to control and the other treatments. Total antioxidant capacity increased in all treated seeds compared with control. The highest antioxidant capacity was detected in the presence of SW (Table 3).

**Table 3.** Total antioxidant activity and total phenol content in quinoa seeds 3 days after sowing with different salt treatments: A= control; B= 100 mM NaCl, 2.54 mM KCl, 2.54 mM CaCl_2_, 13.38 mM MgCl_2_, 25 % SW; C= 200 mM NaCl, 5.08 mM KCl, 5.08 mM CaCl_2_, 26.76 mM MgCl_2_, 50 % SW; *D*= 300 mM NaCl, 7.62 mM KCl, 7.62 mM CaCl_2_, 40.1 mM MgCl_2_, 75 % SW; *E*= 400 mM NaCl, 10.16 mM KCl, 10.16 mM CaCl_2_, 53.52 mM MgCl_2_, 100 % SW. *Different letters in the same column denote significant differences among treatments (*P* ≤ 0.05). Mean ± SE (*n* = 4–5).

	NaCl	KCl	CaCl_2_	MgCl_2_	SW
Total antioxidant activity (µmol α-tocopherol/g FW)					
A	0.65 ± 0.02^c^	0.65 ± 0.02^b^	0.65 ± 0.02^c^	0.65 ± 0.02^c^	0.65 ± 0.02^d^
B	3.15 ± 0.02^a^	3.06 ± 0.04^a^	1.91 ± 0.10^b^	1.95 ± 0.10^b^	2.24 ± 0.08^c^
C	2.98 ± 0.02^a^	2.91 ± 0.09^a^	2.62 ± 0.03^a^	2.69 ± 0.02^a^	4.13 ± 0.15^a^
D	3.06 ± 0.04^a^	2.89 ± 0.1^a^	2.50 ± 0.02^a^	2.52 ± 0.08^a^	3.24 ± 0.03^b^
E	2.44 ± 0.05^b^	2.94 ± 0.05^a^	2.51 ± 0.03^a^	2.75 ± 0.10^a^	3.17 ± 0.02^b^
Total phenols (mg TAET/g DW)					
A	209 ± 10^c^	209 ± 10^a^	209 ± 10^a^	209 ± 10^a^	209 ± 10^c^
B	285 ± 10^b^	200 ± 15^a^	198 ± 10^a^	167 ± 8^b^	555 ± 25^b^
C	307 ± 8^b^	180 ± 10^a^	223 ± 20^a^	169 ± 5^b^	521 ± 10^b^
D	370 ± 12^a^	181 ± 12^b^	223 ± 18^a^	171 ± 10^b^	625 ± 20^a^
E	347 ± 9^a^	180 ± 13^b^	224 ± 22^a^	163 ± 6^b^	568 ± 10^b^

#### Ion contents

**Figure 2.**
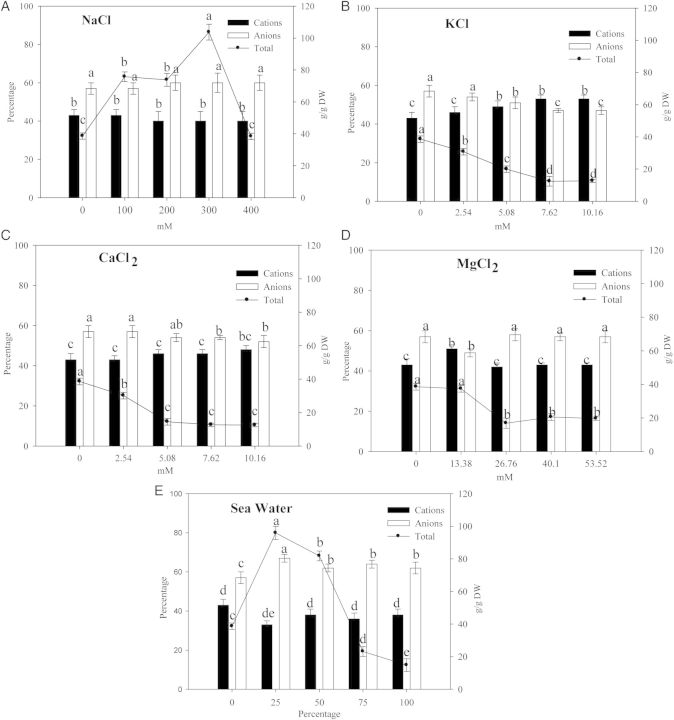
Total ion content, cation and anion percentages in seeds of quinoa after 3 days of different salt treatments. Mean ± SE (*n* = 4–5). Different letters denote significant differences among the treatments (*P* ≤ 0.05).

In seeds 3 days after sowing, the total quantity of ions increased with increasing concentration of NaCl. A similar response was observed in the presence of SW, the only exception being at the higher concentrations (mainly ungerminated seeds) (Fig. 2). In the presence of KCl and CaCl_2_, the total ionic concentration gradually decreased with increasing concentrations of salts due to the increased number of non-germinated seeds (Fig. 2). On increasing MgCl_2_ concentrations, the reduction in total ion concentration compared with control is likely due to the greater seed dry weight observed (+20 %). The ratio of cations/anions was unchanged in CaCl_2_ and MgCl_2_ and in NaCl up to a concentration of 400 mM. Increasing the concentration of KCl caused an increase in cations and a concomitant decrease in anion percentage (Fig. 2). Seawater, at the lowest concentrations (25 and 50 %), increased the total ions, lowering the amount of cations (33 %) with respect to the anions. Conversely, at the highest concentrations (75 and 100 %), SW decreased the number of germinated seeds and consequently the quantity of total ions but did not affect the cation–anion ratio (Fig. 2).

The ratio of Na^+^ to cations and of Cl^−^ to anions changed significantly depending on the salts used (Table 4). The ratio of Na^+^/cations increased significantly in comparison to the control with increasing the concentration of NaCl and SW. No differences were observed in the presence of MgCl_2_, while with CaCl_2_ a slight decline was observed with respect to the control. The greatest significant decrease in Na^+^/cations ratio (ranging from 30 to 22 %) was observed in seeds under KCl treatment. For the Cl^−^/anions ratio, the lowest values were observed in the presence of KCl and the highest with NaCl. Increasing the concentration of SW and NaCl, increased the Na^+^/Cl^−^ ratio with respect to the control, while this ratio decreased in the presence of other salts when their concentrations increased (Table 4). The greatest decrease in K^+^/Cl^−^ ratio was observed in the presence of NaCl with a reduction ranging from 49 to 87 %. Mg^2+^/Cl^−^ and NH_4_^+^/Cl^−^ ratios decreased with respect to the control, mainly with increasing salt concentrations (Table 4). The Ca^2+^/Cl^−^ ratio decreased in each treatment except for CaCl_2_ and KCl. The PO_4_^3−^/Cl^−^ ratio was significantly reduced compared with control in the presence of SW, NaCl and MgCl_2_ (Table 4). The highest SO_4_^2−^/Cl^−^ ratios were observed in the presence of SW and the lowest under NaCl treatment.

**Table 4.** Cation and anion content against chloride, in seeds of quinoa treated with different salts, expressed as percentages. *Different letters in the same column denote significant differences among treatments (*P* ≤ 0.05). Mean ± SE (*n* = 4–5).

Treatments		Na^+^/cations	Cl^−^/anions	Na^+^/Cl^−^	PO_4_^3−^/Cl^−^	SO_4_^2−^/Cl^−^	K^+^/Cl^−^	NH_4_^+^/Cl^−^	Mg^2+^/Cl^−^	Ca^2+^/Cl^−^
Control		5.0^d^	13^e^	29^d^	635^a^	23^b^	349^b^	76^a^	69^a^	28^b^
NaCl	100 mM	41^c^	49^c^	63^b^	102^d^	1.8^d^	51^d^	23^c^	15^d^	3.2^d^
NaCl	400 mM	72^a^	64^b^	76^a^	54^e^	0^d^	13^e^	5.8^e^	10^d^	1.9^d^
MgCl_2_	13.38 mM	5.0^d^	33^d^	16^e^	202^c^	6.5^c^	185^c^	34^b^	53^a^	19^b^
MgCl_2_	53.52 mM	5.0^d^	63^a^	6.1^f^	58^e^	0^d^	53^d^	9.6^d^	44^b^	6.8^c^
CaCl_2_	2.54 mM	6.0^d^	16^e^	30^d^	530^b^	15^b^	345^b^	45^b^	55^a^	14^c^
CaCl_2_	10.16 mM	4.3^d^	37^d^	11^e^	170^d^	0.82^d^	130^c^	15^d^	43^b^	43^a^
KCl	2.54 mM	3.5^d^	13^e^	24^d^	500^b^	100^a^	485^a^	7.4^a^	62^a^	29^b^
KCl	10.16 mM	3.2^d^	45^c^	18^e^	120^d^	2.6^c^	273^b^	7.3^e^	29^c^	45^a^
SW	25 %	37^c^	35^d^	51^c^	58^e^	128^a^	51^d^	13^d^	14^d^	9.3^c^
SW	100 %	55^b^	57^b^	63^b^	41^f^	19^b^	36^d^	0^f^	13^d^	3.2^d^

### Experiment 2: morphological, physiological and biochemical responses of seedlings

#### Growth parameters

Seawater and NaCl, at the highest concentrations, affected the dry weights of the whole seedlings, as shown by the highest fresh weight/dry weight (FW/DW) ratio (Table 5), and additionally they reduced the root mass ratio (RMR). These findings suggest that the reduction of root mass may be the cause of the decrease in the total dry matter of the seedlings (Table 5). Investigating the root morphology showed that the total root length in all treatments was the most affected root parameter, as shown by *F*-ratios (Table 6). The plants irrigated with SW (50 %) had root lengths, surface areas and root volumes significantly lower than control (Table 6).

**Table 5.** Total FW/DW ratio, LMR (leaf mass ratio = leaf dry weight/plant dry weight) and RMR (root mass ratio = root dry weight/plant dry weight) of quinoa seedlings after 21 days under different salt treatments. Different letters in the same column denote significant differences among treatments (*P* ≤ 0.05). Mean ± SE (*n* = 4–5).

		FW/DW (g plant^−1^)	LMR (g plant^−1^)	RMR (g plant^−1^)
Control		9.7 ± 0.2^b^	0.81 ± 0.02^b^	0.19 ± 0.01^a^
SW	50 %	11.8 ± 0.2^a^	0.89 ± 0.01^a^	0.11 ± 0.02^b^
KCl	5.08 mM	8.3 ± 0.7^b^	0.83 ± 0.02^b^	0.17 ± 0.01^a^
KCl	10.16 mM	8.5 ± 0.4^b^	0.84 ± 0.02^b^	0.16 ± 0.01^a^
CaCl_2_	5.08 mM	9.0 ± 0.5^b^	0.82 ± 0.03^b^	0.18 ± 0.02^a^
CaCl_2_	10.16 mM	8.8 ± 0.3^b^	0.83 ± 0.01^b^	0.17 ± 0.01^a^
NaCl	200 mM	9.8 ± 0.2^b^	0.82 ± 0.02^b^	0.18 ± 0.02^a^
NaCl	400 mM	10.8 ± 0.2^a^	0.90 ± 0.02 ^a^	0.10 ± 0.01^b^
MgCl_2_	26.76 mM	9.2 ± 0.3^b^	0.78 ± 0.02^b^	0.22 ± 0.03^a^
MgCl_2_	53.52 mM	9.5 ± 0.5^b^	0.82 ± 0.01^b^	0.18 ± 0.02^a^

**Table 6.** Analysis of variance of the effect of different salt treatments on root morphology parameters of quinoa seedlings 21 days old. ***P < 0.001; **P < 0.01; *P < 0.05.

Treatment	Total root length	Surface area	Volume	
SW	2309.20***	200.82***	132.25***	*F*-ratio
	0.99	0.99	0.98	*R*
KCl	56.11***	2.98	8.22*	*F*-ratio
	0.97	0.71	0.86	*R*
CaCl_2_	49.18***	27.73**	8.33*	*F*-ratio
	0.97	0.95	0.86	*R*
MgCl_2_	95.77***	21.25**	11.27**	*F*-ratio
	0.98	0.94	0.89	*R*
NaCl	42.67***	3.94^.^	7.00*	*F*-ratio
	0.97	0.75	0.84	*R*

#### Root parameters

Root length to mass ratio (SRL) and root fineness (RF), under SW, were not different from control while the ratio of root mass to volume (RTD) was lower. In seedlings irrigated with 400 mM NaCl, a higher SRL value indicated longer roots per unit root mass, while RTD and RF ratios were significantly reduced (Table 7), suggesting a decrease in root length and dry weight of seedlings treated with NaCl (200 mM) or MgCl_2_ (26, 76 mM). Root morphology parameters were significantly changed by CaCl_2_ and KCl compared with control but to different extents, depending on salt type (Table 7). NaCl, MgCl_2_ and CaCl_2_, at lower concentrations, significantly increased RTD and RF ratios. No differences were observed when CaCl_2_ and NaCl concentrations increased (Table 7). KCl, at all concentrations, significantly increased RTD and RF ratios, inducing a root system with thinner roots in comparison with control.

**Table 7.** Specific root length (SRL = root length/root DW), root tissue density (RTD = root DW/root volume), root fineness (RF = root length/root volume) of quinoa seedlings after 21 days of different salt treatments. ***P < 0.001; **P < 0.01; *P < 0.05.

		SRL (cm/mg DW)	RTD (mg DW/cm^3^)	RF (cm/cm^3^)
Control		18.7	28	590
SW	50 %	21	23.1*	613
SW	100 %	–	–	–
KCl	5.08 mM	19.3	35**	675*
KCl	10.16 mM	21	31.3*	690**
CaCl_2_	5.08 mM	18.6	31.2*	687**
CaCl_2_	10.16 mM	20.0	27.5	520.9*
MgCl_2_	26.76 mM	16.8	33.4*	636*
MgCl_2_	53.52 mM	20	29.0	601
NaCl	200 mM	18.35	36.6*	659*
NaCl	400 mM	24*	19.6**	522*

#### Ion contents

**Figure 3.**
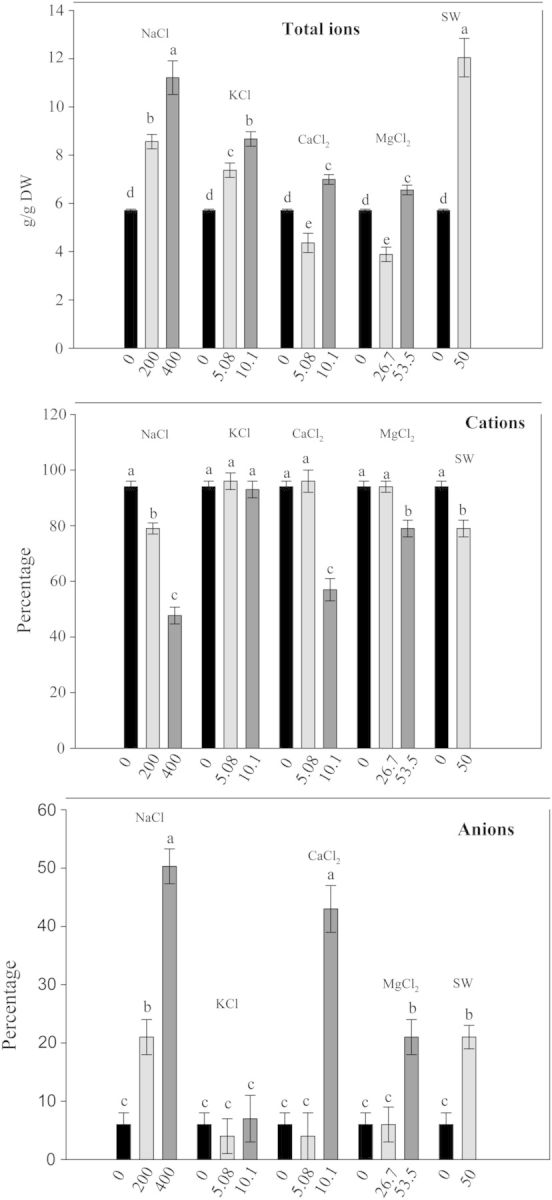
Total ion content, cation and anion percentages in quinoa seedlings after 21 days of different salt treatments. Mean ± SE (*n* = 4–5). Different letters denote significant differences among the treatments (*P* ≤ 0.05).

In 21-day-old seedlings, total percentage of ions increased in the presence of NaCl, SW and KCl at all concentrations (Fig. 3) and at the highest concentrations of CaCl_2_ and MgCl_2_. Total cations (Fig. 3) decreased in the presence of NaCl at all concentrations and at the highest concentrations of SW, MgCl_2_ and CaCl_2_, with a concomitant increase in anion percentages (Fig. 3). No significant differences, in comparison to control, were observed in the presence of KCl.

**Figure 4.**
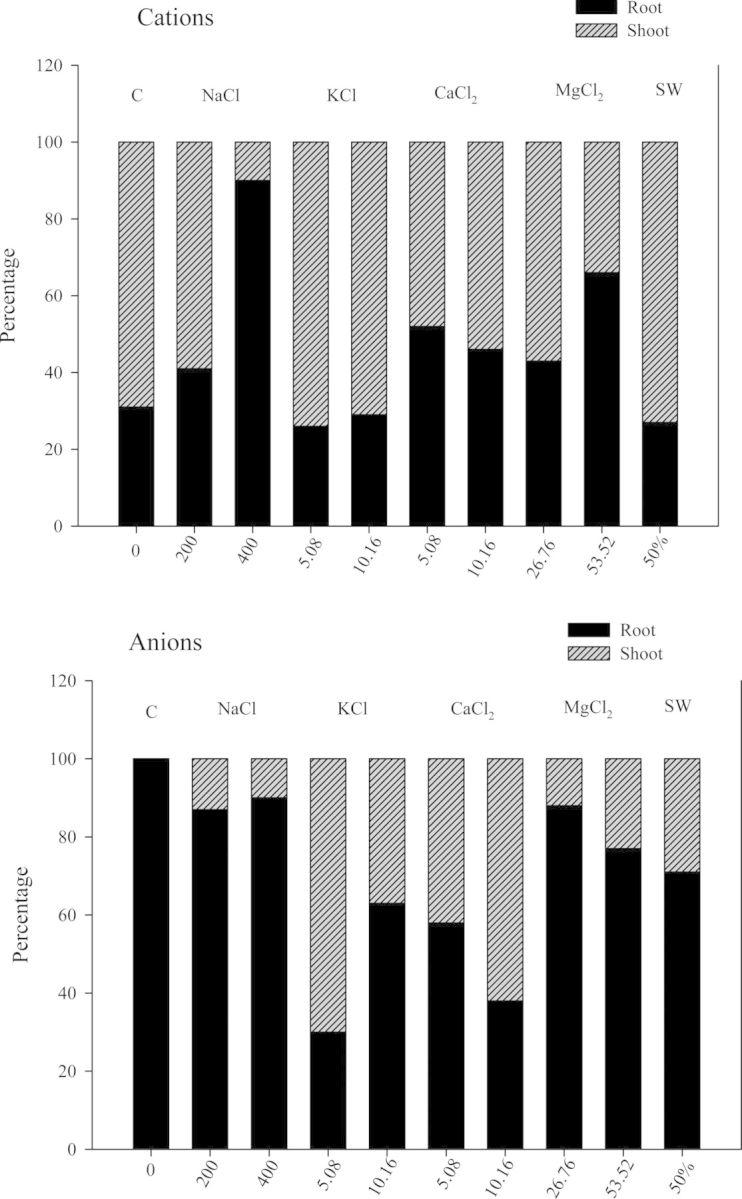
Cation and anion percentages in root and shoot of quinoa seedlings after 21 days of different salt treatments.

Different salts caused a different distribution of cations and anions between root and shoot (Fig. 4). More cations were accumulated in shoots than in roots, decreasing in shoots when NaCl and MgCl_2_ concentrations increased, while roots accumulated more anions than cations. The highest accumulation of anions was observed with CaCl_2_ and KCl but with a different trend. In CaCl_2_, the anions increased in a concentration-dependent manner; in contrast increasing KCl concentrations lowered the anion percentage (Fig. 4). NaCl and MgCl_2_ increased the cation concentration in roots as their external concentrations increased (Fig. 4).

The ratios of Na^+^/total cations and of Cl^−^/anions changed significantly depending on the salts used (Table 8). The Na^+^/cations ratio increased in comparison to the control with increasing the concentration of NaCl and SW. In contrast, Na^+^/cations ratio decreased with increasing the concentration of KCl, MgCl_2_ and CaCl_2_. Cl^−^/anions ratios increased in the different salts at all concentrations, the highest value being observed with NaCl treatment. Increasing the concentration of SW and NaCl increased the Na^+^/Cl^−^ ratio, while it was lowered in the other salts as their concentration increased. The K^+^/Cl^−^ ratio decreased in the presence of all salts except for KCl, the greatest decrease being observed in NaCl. The Mg^2+^/Cl^−^ ratio decreased with increasing concentrations of salts, other than for MgCl_2_. A similar situation was seen for the Ca^2+^/Cl^−^ ratio, which decreased in each treatment except for CaCl_2_. The NH_4_^+^/Cl^−^ ratio decreased in all situations as did SO_4_^2−^/Cl^−^ ratios, where the highest values were detected in SW (Table 8).

**Table 8.** Cation and anion content against chloride, in seedlings of quinoa treated with different salts, expressed as percentages. *Different letters in the same column denote significant differences among treatments (*P* ≤ 0.05). The values correspond to the average of five replicates.

Treatments		Na^+^/cations	Cl^−^/anions	SO_4_^2−^/Cl^−^	K^+^/Cl^−^	NH_4_^+^/Cl^−^	Mg^2+^/Cl^−^	Ca^2+^/Cl^−^	Na^+^/Cl^−^
Control		15^d^	–	–	–	–	–	–	–
NaCl	200 mM	55^b^	83^a^	18^b^	18^d^	3^e^	15^d^	24^e^	1.9^g^
NaCl	400 mM	65^a^	87^a^	15^b^	11^e^	2.9^e^	5^e^	21^e^	1.2^g^
MgCl_2_	26.73 mM	17^d^	41^d^	19^b^	26^cd^	36^b^	46^b^	63^c^	62^c^
MgCl_2_	53.46 mM	11^e^	85^a^	18^b^	21^d^	18^c^	49^b^	50^d^	4^f^
CaCl_2_	5.08 mM	9^e^	42^d^	16^b^	37^c^	54^a^	23^c^	70^b^	110^b^
CaCl_2_	10.16 mM	2.6^f^	78^b^	4.7^c^	13^e^	20^c^	9^e^	78^a^	149^a^
KCl	5.08 mM	12^d^	13^e^	6.4^c^	345^b^	14^d^	23^a^	56^d^	27^d^
KCl	10.16 mM	6^e^	71^c^	4.0^c^	654^a^	5^e^	21^c^	52^d^	26^d^
SW	50 %	43^c^	67^c^	48^a^	30^c^	1.9^f^	54^b^	11^f^	11^e^

#### Enzyme activities, phenols and antioxidants

**Figure 5.**
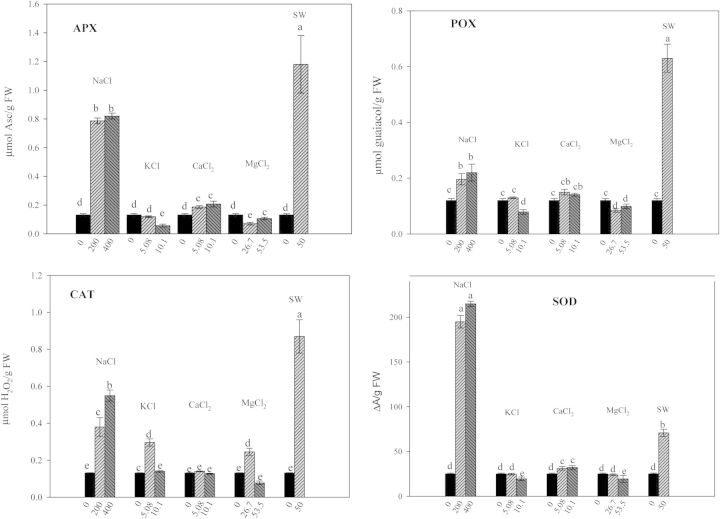
Effect of different salts on antioxidant enzymatic activities of quinoa seedlings after 21 days of different salt treatments. Mean ± SE (*n* = 4–5). Different letters denote significant differences among the treatments (*P* ≤ 0.05).

The activity of the antioxidant enzymes depended on the salt and on the concentrations used (Fig. 5). Ascorbate peroxidase activity significantly decreased in the presence of MgCl_2_ and KCl. In contrast, it increased in CaCl_2_-, SW- and NaCl-treated seedlings compared with control. POX activity increased in all treatments except for MgCl_2_ and KCl. The most significant increase in catalase activity was in NaCl and SW. The same trend was observed for the SOD activity, with the highest values seen in the presence of SW and NaCl.

A significant increase in total phenols was observed in seedlings grown with NaCl and SW (Table 9). The SW was the most damaging agent, causing a 2-fold increase in the concentration of phenols. The total antioxidant capacity was doubled by NaCl and tripled by SW in respect to the control (Table 9).

**Table 9.** Total antioxidant activity and total phenol content in quinoa seedlings after 21 days with different salt treatments: A = 200 mM NaCl, 5.08 mM KCl, 5.08 mM CaCl_2_, 26.76 mM MgCl_2_, 50 % SW; B = 400 mM NaCl, 10.16 mM KCl, 10.16 mM CaCl_2_, 53.52 mM MgCl_2_, 100 % SW. Different letters in the same column denote significant differences among treatments (*P* ≤ 0.05). Mean ± SE (*n* = 4–5).

	NaCl	KCl	CaCl_2_	MgCl_2_	SW
Total antioxidant activity (mmol α-tocopherol/g FW)					
Control	2.07 ± 0.03^c^	2.07 ± 0.03^a^	2.07 ± 0.03^a^	2.07 ± 0.03^a^	2.07 ± 0.03^b^
A	4.35 ± 0.05^b^	1.76 ± 0.02^b^	2.06 ± 0.02^a^	2.16 ± 0.07^a^	6.20 ± 0.03^a^
B	6.01 ± 0.06^a^	1.56 ± 0.02^c^	1.82 ± 0.04^b^	1.58 ± 0.04^b^	–
Total phenols (mg TAET/g DW)					
Control	272 ± 40^c^	272 ± 40^a^	272 ± 40^a^	272 ± 40^a^	272 ± 40^b^
A	635 ± 20^b^	322 ± 30^a^	252 ± 30^a^	222 ± 30^a^	1075^a^
B	840 ± 30^a^	312 ± 10^a^	240 ± 20^a^	239 ± 20^a^	–

## Discussion

In the Mediterranean region, besides water scarcity or high coastal soil salinity, it is mainly where saline water is used for irrigation that adverse effects are seen on crops, delaying or preventing germination and seedling growth (Hegarty 1978; Almodares *et al.* 2007). Utilization of halophytes as crops would help in highly salinized zones, where only poor quality water, unsuitable for most agriculture, is available (Rozema and Flowers 2008).

In this context, quinoa a facultative halophyte with exceptional nutritional quality could be useful to recover salinized land and to increase the poor agricultural economy of semiarid regions of the Mediterranean area. Our study focused on germination and seedling growth, because crop establishment depends on a successful germination and seedling emergence. Optimal germination for most halophytes has been reported in non-saline conditions (Khan *et al.* 2002; Gul *et al.* 2013), and our data conform to these findings, showing toxicity of different salts. Results provided evidence for the existence of both ionic and osmotic effects by different treatments on seeds, depending on the salts used.

Our data clearly demonstrated that SW was the most detrimental solution affecting seed germination and seedling emergence of quinoa, mainly through its IE, confirming previous work showing that germination of halophytes was inhibited more by SW than different chlorides of Na, K, Mg (Joshi *et al.* 1995). There is little information available on comparative influence of single salts and SW on seed germination of other halophytes (Joshi *et al.* 1995; Baskin and Baskin 1998; Houle *et al.* 2001; Zia and Khan 2002; Atia *et al.* 2006; Liu *et al.* 2006). Some authors found NaCl more detrimental than SW and others the opposite (Tirmizi *et al.* 1993; Zia and Khan 2002; Duan *et al.* 2003). Our data showed that the inhibition of different salt solutions on seed germination was in the order of SW > NaCl > KCl > CaCl_2_ > MgCl_2_ with no significant differences among the treatments in germination rapidity, except for the SW. The greatest negative effects of SW may be due to ion toxicity on germination, as a consequence of a coincident increase in cations and anions. Ion toxicity during germination has been previously demonstrated by Zehra *et al.* (2013) for the halophytic reed *Phragmites karka*: the inhibitory effect of different salts was interpreted mainly as an IE.

Although NaCl is the predominant salt in SW, its effects on seed germination and seedling growth were less detrimental than SW itself. The negative effects of SW on seedling growth may be ascribed to the induced accumulation of SO_4_^2−^ (7.67 mmol g^−1^ DW, at least five times more than the other treatments) in leaves and of SO_4_^2−^ (0.88 mmol g^−1^ DW) and Cl^−^ (47.97 mmol g^−1^ DW) in roots. Sulfate is one of the components of sulfur-containing amino acids (cysteine and methionine) and many other compounds (e.g. glutathione or ferredoxin), which play important physiological functions, but when SO_4_^2−^ is present in high concentration, it may affect plant development and crop yield, becoming injurious to plants (Lianes *et al.* 2013). Lianes *et al.* (2013) previously showed that when the SO_4_^2−^ is present in the medium, the capacities for ion compartmentalization and osmotic adjustment were reduced in the halophyte *Prosopis strombulifera*, resulting in water imbalance and symptoms of toxicity due to altered carbon metabolism (e.g. synthesis of sorbitol instead of mannitol, reduced sucrose production and protein content). This inhibition was partially mitigated when SO_4_^2−^ and Cl^−^ were present together in the solution, demonstrating a detrimental effect of the sulphate ion on plant growth (Reginato *et al.* 2013).

According to Munns (2002), the time scales for the osmotic and specific ionic component of salinity stress differ significantly, with the osmotic component dominating the first several days. Interestingly, however, comparing seed germination and seedling growth in the different salts, the results suggest that most probably ion toxicity is more detrimental to seedlings compared with the osmotic component of salt stress, as evidenced by the effect of SW treatment. This high salinity tolerance of quinoa, during germination and early seedling growth, may be explained by the existence of a significant gradient in the accumulation of potentially toxic (Na and Cl) and non-toxic essential (K, Mg, Ca, P and S) elements in seeds and also in the different distribution between shoot and root in salt-treated seedlings, as already demonstrated by Koyro and Eisa (2008). Hence, we suggest that, once the seed's ability to exclude toxic Na^+^ from the developing embryo fails, ion toxicity occurs, and seeds become unviable. The details of the distributions of ions between root and shoot showed differences among treatments; specifically with NaCl in shoot, we observed a significant accumulation of Na^+^, and little Cl^−^. In accordance with previous investigations (Eisa *et al.* 2000), Na^+^ was shown to be preferentially accumulated in shoots thereby the plants avoid excessive ion accumulation in the root tissues (Koyro 2000; Ashraf *et al.* 2006).

Seawater caused an accumulation of Na^+^ and SO_4_^2−^ both in roots and in shoots, and an accumulation of Cl^−^ in roots. Excessive accumulation of ions in halophytes (salt includers) under high substrate salinities (such a full strength SW) can lead to toxic effects in plants (Munns 2005). The cause of injury is probably the salt load exceeding the ability of cells to compartmentalize salts in the vacuole. Salts might then build up rapidly in the cytoplasm inhibiting enzyme activity or alternatively, they might build up in cell walls, dehydrating the cell.

Considering the high energy cost of *de novo* synthesis of organic osmolytes (Raven 1985), we can suppose that the seedlings tend to use Na^+^ for osmotic adjustment. Hariadi *et al.* (2011) previously showed in quinoa that accumulation of Na^+^ and K^+^ was responsible for >95 % of cell turgor in old leaves and between 80 and 100 % in young leaves. A further role in the maintenance of turgor was also attributed to Cl^−^ accumulated in roots (James *et al.* 2006). Our results showed that the Cl^−^ concentration was more than enough to contribute to osmotic adjustment maintaining root turgor as previously demonstrated in seedling of *Stylosanthes guianensis* by Veraplakorn *et al.* (2013). Thus, it appears that the better germination and growth of cv. Titicaca observed in NaCl with respect to the other salts and SW may be achieved by the accumulation of inorganic osmolytes, particularly of Na^+^ in shoots, and of Cl^−^ in roots. The differences in ion uptake and distribution may be ascribed to properties of the roots. Roots have a high degree of plasticity, enabling plants to cope with a wide range of soil constraints (Ho *et al.* 2005; Panuccio *et al.* 2011). Root morphology is a compromise among costs of resource capture, transport and efficiency (Malamy 2005). Some morphological modifications at the individual root level can affect the structural and physiological characteristics of the entire root system and this can change water uptake and nutrient supply by plants. Specific root length, indicating root functionality (Ryser 2006), characterizes the economic aspects of a root system, defining the cost-benefit ratio. Generally, under high salinity the costs per root length is minimized because of the growth limiting conditions. SW (50 %) reduced root growth and elongation, suggesting a decrease in photosynthate supply from the shoot. At the highest NaCl concentration, the greatest SRL ratio suggests the plants maximized the effectiveness of roots in water and nutrient uptake (Fitter 1991). At the lowest concentrations of NaCl, KCl, CaCl_2_ and MgCl_2_, the high root tissue density and root fineness ratios indicated that the seedlings explored a larger soil volume per unit of root surface area under stress than in its absence. In short, our data suggest that root morphology modifications should not be considered as a simple growth reduction, but rather as an induced reorientation of growth to avoid stress.

The results of this study clearly indicated that salt tolerance in this variety of quinoa is largely conferred by a delicate balance between osmotic adjustment and ion accumulation, showing differences in the ion compartmentalization between root and shoot. The greater negative effect of SW compared with NaCl, MgCl_2_ CaCl_2_ and KCl used separately suggests an additive and/or an interactive effect of these salts which cause an accumulation of ions in excess or leading to ion toxicity.

## Conclusions

In conclusion, the present findings allow us to speculate that quinoa cv. Titicaca is a NaCl-tolerant cultivar of quinoa. Osmotic adjustment to NaCl salinity is largely conferred by inorganic ions, especially Na^+^, the main osmoregulatory material in the seedlings. The high SRL contributed to a high relative NaCl salinity tolerance in Titicaca, maintaining water and nutrient uptake. Higher SW toxicity may have been caused by SO_4_^2−^ accumulation in seedlings that affected Titicaca germination and growth more than Cl^−^. Even if salinity reduced the productivity in terms of biomass, there was an increase in the antioxidant compounds, important health-protecting factors in food. On the basis of salt soil classifications currently used in all countries of the world, our results suggest that saline-sodic soils may be suitable for the cultivation of quinoa.

## Sources of Funding

The research in the Mediterranea University laboratory and travelling was funded by Fattoria della Piana Company and by COST (STSM FA0901).

## Contributions by the Authors

S.S.A. participated in the experiments, M.R.P. and A.M. did the experiments, analysed the data and wrote the manuscript, S.E.J. participated in the writing of the manuscript, acquired the funds for S.S.A. through the COST action ‘Putting Halophytes to Work’, and provided quinoa seed material for the study.

## Conflicts of Interest Statement

None declared.
